# Supporting data on combined transcriptomic and phosphoproteomic analysis of BMP4 signaling in human embryonic stem cells

**DOI:** 10.1016/j.dib.2021.106844

**Published:** 2021-02-06

**Authors:** Angelos Papadopoulos, Varvara Chalmantzi, Marko Hyvönen, Dimitris Stellas, Marika Syrrou, Theodore Fotsis, Carol Murphy

**Affiliations:** a School of Biosciences, College of Life and Environmental Sciences, University of Birmingham, Edgbaston, Birmingham, B15 2TT, United Kingdom; b Cardiovascular Division, King's College London British Heart Foundation Centre of Excellence, London, SE5 9NU, United Kingdom; c Department of Biochemistry, University of Cambridge, United Kingdom; d Institute of Chemical Biology, National Hellenic Research Foundation, 11635 Athens, Greece; e Department of Biomedical Research, Institute of Molecular Biology and Biotechnology, Foundation of Research and Technology-Hellas, University Campus of Ioannina, 45110 Ioannina, Greece; f Laboratory of Biology, Medical School, University of Ioannina, 45110 Ioannina, Greece; g Laboratory of Biological Chemistry, Medical School, University of Ioannina, 45110 Ioannina, Greece

**Keywords:** Human embryonic stem cells, Activin A, TGFβ, mTeSR

## Abstract

Human embryonic stem cells exhibit great potential as a therapeutic tool in regenerative medicine due to their self-renewal and trilineage differentiation capacity. Maintaining this unique cellular state has been shown to rely primarily on the Activin A / TGFβ signaling pathway. While most conventional culture media are supplemented with TGFβ, in the current study we utilize a modified version of the commercially available mTeSR1, substituting TGFβ for Activin A in order to preserve pluripotency. (1) Cells cultured in ActA-mTesR express pluripotency factors NANOG, OCT4 and SOX2 at comparable levels with cells cultured in TGFβ-mTeSR. (2) ActA-mTeSR cultured cells retain a physiological karyotype. (3) Cells in ActA-mTeSR maintain their trilineage differentiation capacity as shown in the teratoma formation assay. This system can be used to dissect the role of Activin A, downstream effectors and signaling cascades in human embryonic stem cell responses.

## Specifications Table

**Table:** 

Subject	Cell Biology
Specific subject area	Human Embryonic Stem Cells
Type of data	Graph, Figure
How data were acquired	Image capture (Leica TCS SP8 and SP5 confocal microscopes, LASAF software) Western blotting (LI-COR Odyssey imaging system, image studio software) Data analysis (GraphPad Prism version 6.0) qRT-PCR (Light Cycler) Teratoma formation assay (H&E staining)
Data format	Raw, Analyzed
Parameters for data collection	H1 cells were cultured in TGFβ or Activin A- based mTeSR1 for several passages were tested for expression of pluripotency markers, karyotypic abnormalities as well as for *in vitro* and *in vivo* differentiation capacity.
Description of data collection	Pluripotency assessment was performed by indirect immunofluorescence of H1 colonies with antibodies against NANOG, OCT4 and SOX2. *In vitro* differentiation potential was assessed by subjecting H1 cells to three separate protocols for mesoderm, endoderm and ectoderm differentiation. Karyotypic analysis was performed by colchicine-induced mitotic arrest and Giemsa staining. Assessing *in vivo* differentiation potential required the formation of teratomas in NOD/SCID mice and the subsequent H&E staining.
Data source location	University of Birmingham, School of Biosciences, Birmingham, United Kingdom
Data accessibility	Papadopoulos, Angelos (2021), “Supporting data on Combined transcriptomic and phosphoproteomic analysis of BMP4 signaling in human embryonic stem cells”, Mendeley Data, V1, https://doi.org/10.17632/tsk65tw2kx.1
Related research article	[1] A. Papadopoulos et al., “Combined transcriptomic and phosphoproteomic analysis of BMP4 signaling in human embryonic stem cells.,” *Stem Cell Res.*, vol. 50, no. November 2020, p. 102,133, Dec. 2020, https://doi.org/10.1016/j.scr.2020.102133.

## Value of the Data

• These data provide an Activin A dependent culture system substituting TGFβ in mTeSR1 and capable of maintaining the pluripotency of hESCs.
• The culture system developed is a versatile tool for studying signaling pathways in hESCs.
• This system allows for complete control over the growth factors and compounds included in a standard hESC culture and can therefore be employed to develop novel pluripotency maintenance or differentiation protocols.

## 1 Data Description

**Fig. 1:**
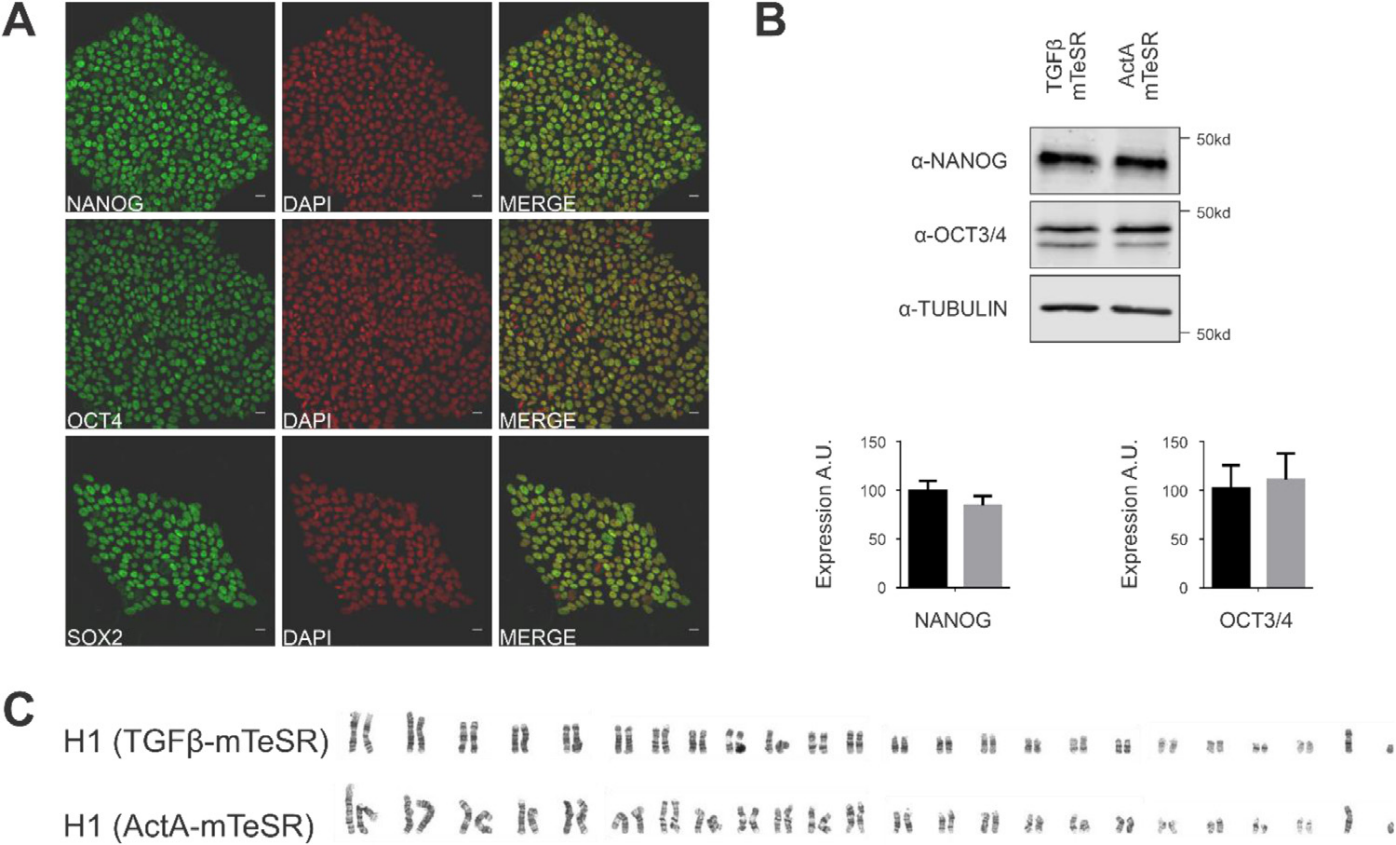
H1 cells cultured in ActA-mTeSR express pluripotency markers and maintain a normal karyotype. **(A)** H1 cells cultured in ActA-mTeSR immunostained with α-NANOG, α-OCT4 and α-SOX2 antibodies. Scale bar = 10 μm. **(B)** H1 cells cultured in TGFβ- (control) or ActA-mTeSR were lysed and immunoblotted with α-NANOG, α-OCT4 and α-TUBULIN antibodies. Expression levels of NANOG and OCT3/4 were quantified by densitometry using TUBULIN as loading control (*N* = 3). **(C)** H1 cells cultured in TGFβ- (control) or ActA-mTeSR were arrested in metaphasis, lysed and the metaphasic chromosomes were extracted and stained with Giemsa reagent.

To develop an mTeSR-dependent human embryonic stem cell (hESC) culture method replacing TGFβ with Activin A, H1 cells were cultured in mTeSR without select factors supplemented with LiCl, GABA, pipecolic acid and FGF2 at the concentrations stated in the manufacturer's protocol [2]. Media was also supplemented with 0.6 ng/ml TGFβ (TGFβ-mTeSR) or 0.5 ng/ml Activin A (ActA-mTeSR). After adapting cells in ActA-mTeSR for 10 passages, localization of the pluripotency factors NANOG, OCT4 and SOX2 was assessed by immunofluorescence (Fig. 1A), protein levels of NANOG and OCT4 were quantified by western blotting (Fig. 1B) and the karyotypic profile was inspected by Giemsa staining (Fig. 1C).

**Fig. 2:**
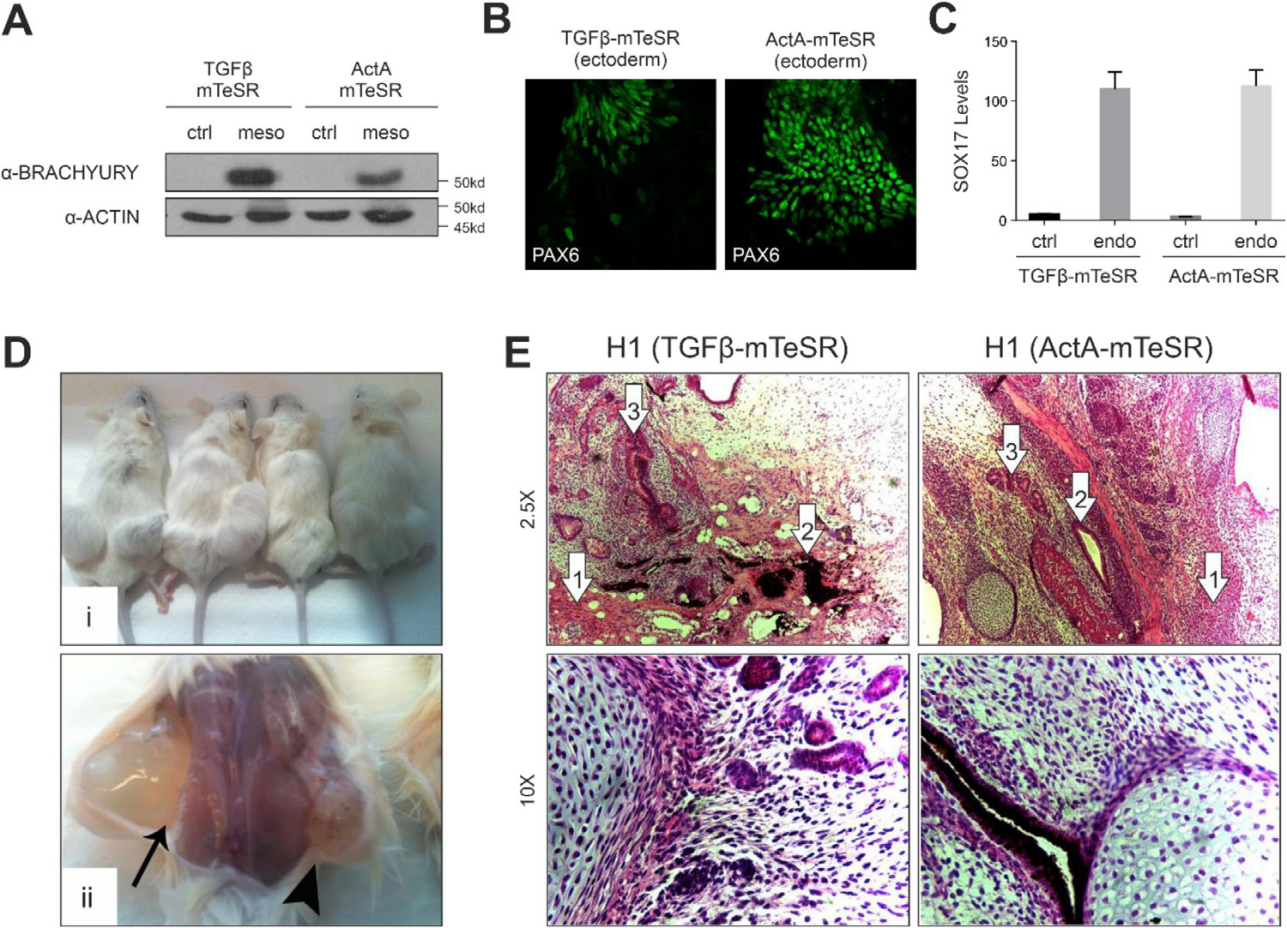
H1 cells cultured in ActA-mTeSR retain their capacity for trilineage differentiation and *in vivo* teratoma formation. **(A)** H1 cells cultured in TGFβ- (control) or ActA-mTeSR were differentiated towards mesoderm for 72 hrs, lysed and immunoblotted with α-BRACHYURY and α-ACTIN antibodies. **(B)** H1 cells cultured in TGFβ- (control) or ActA-mTeSR were differentiated towards ectoderm following an embryoid body formation assay and immunostained with α-PAX6. **(C)** H1 cells cultured in TGFβ- (control) or ActA-mTeSR were subjected to endodermal differentiation for 5 days. Total RNA was extracted and used to quantify SOX17 transcription levels by qPCR. **(Di)** Teratomas formed in 4 separate NOD/SCID mice by subcutaneous injections of H1 cells cultured in TGFβ-mTeSR (left flank) or ActA-mTeSR (right flank). **(Dii)** TGFβ-mTeSR (arrow) and ActA-mTeSR (arrowhead) cultured H1 cells were injected into the same animals to limit subject variability. **(E)** H&E-stained teratoma sections. Numbered arrows denote different cell types observed: (1) cartilage, (2) melanin producing epithelial cells and (3) small glandular formations.

Subsequently, ActA-mTeSR-adapted cells were assessed for their *in vitro* trilineage differentiation capacity. For mesodermal specification, H1 cells were induced with Activin A and BMP4. BRACHYURY levels were analyzed by Western blotting (Fig. 2A). For ectodermal differentiation, H1 cells were subjected to an embryoid body formation assay and stained for PAX6 (Fig. 2B). For endodermal differentiation, H1 cells were induced with Activin A in a serum-containing medium and transcription levels of SOX17 were evaluated by qPCR (Fig. 2C).

Last, ActA-mTeSR cultured cells were assessed for their capacity to form *in vivo* teratomas. Cells cultured in regular TGFβ-mTeSR were used as a positive control. Teratoma size differences between the two cell groups were not scored for, as this methodology was applied in a qualitative manner as a pluripotency test (Fig. 2D). Haematoxylin and eosin staining was applied to visualize the different cell types that emerged (Fig. 2E).

Activin A was first implicated in pluripotency when its precursor was found in the secretome of mouse embryonic fibroblasts (MEFs) which served as feeders for hESC cultures [3]. Since then, several Activin A-dependent systems have been developed for the undifferentiated propagation of hESCs bypassing the need for MEFs or MEF secreted factors [4,5]. Our study provides an additional versatile Activin A-dependent medium which allows for the study of pluripotency and differentiation in a chemically defined background.

## 2 Experimental Design, Materials and Methods

### 2.1 Cell culture conditions

The matrigel-adapted hESC line H1 was purchased from WiCell (WB0113; Madison, WI, USA) and maintained in mTeSR without select factors (05,896; Stem Cell Technologies) supplemented with 1 mM lithium chloride (62,476; Sigma), 1 mM G-aminobutyric acid (A5835; Sigma), 0.1 mM pipecolic acid (P2519; Sigma), 100 ng/mL FGF2 (11,343,627; ImmunoTools) and 0,5 ng/mL Activin A (Dr Marko Hyvönen, University of Cambridge) or 0.6 ng/mL TGFβ (100–21; Peprotech) on matrigel (354,277; Corning) coated dishes. H1 cells were cultured at 37 °C in a humidified incubator containing 5% CO_2_.

### 2.2 Immunofluorescence assays

Sub-confluent H1 cells on 35 mm μ-dishes (IB81151; Ibidi) were subjected to immunofluorescence as previously described [6]. Cells were incubated with primary antibodies against NANOG (3580 s; Cell Signaling), OCT3/4 (sc-5279; Santa Cruz Biotechnology), SOX2 (MAB2018; R&D) and PAX6 (DHSB). Subsequently, samples were stained with Alexa Fluor®488 α-Rabbit IgG (711–545–152; Jackson ImmunoResearch), Alexa Fluor®594 α-Mouse IgG (715–585–151; Jackson ImmunoResearch) or FITC a-Mouse (715–095–151; Jackson ImmunoResearch) and DAPI (D9542; Sigma). Samples were imaged using a Leica TCS SP8 or SP5 confocal microscope with a 40X/1.30 NA or 10X objective, and LASAF Imaging Software. All Images are maximum projections.

### 2.3 Western blot analysis

For western blotting, total cell lysates were electrophoresed and transferred onto nitrocellulose membranes (10,600,012; GE Healthcare) which were incubated with primary antibodies against NANOG, OCT3/4 and TUBULIN (E7; DSHB). Next, membranes were stained with near-infrared fluorescent secondary antibodies IRDye® 800CW α-Rabbit IgG (926–32,213; LI-COR) and IRDye® 680RD α-Mouse IgG (926–68,070; LI-COR). Results were visualized on a LI-COR Odyssey imaging system. Densitometry was carried out using the Image Studio Lite software. For Brachyury expression membranes were incubated with primary antibodies against BRACHYURY (R&D; AF2085) and ACTIN (EMD Millipore; MAB1501). For protein visualization, membranes were incubated for 1 min with Amersham ECL Western Blotting Detection Reagent (Amersham; RPN2209) and exposed onto Fuji Super Rx X-ray films.

### 2.4 Quantitative real-time PCR

Total RNA was extracted according to the manufacturer's protocol (Macherey Nagel; 119–22,402). Sample quantity and purity was determined using Nanodrop (ND-1000 V3.8.1) and samples were aliquoted and stored in −80 °C. qPCR reactions were prepared using the QuantiTect SYBR® Green RT-PCR Kit (Qiagen; 204,243) and appropriate primer pairs to a final concentration of 3 μM. The reaction was performed in a Roche LightCycler PCR System. CT values were normalized to GAPDH using the equation: 2^-ΔCt. Primer sequences: SOX17 (FW:TCCCATGCACCCCCGACTC, RV:TGCTGGTGCTGGTGCTGGTGTTG), GAPDH (FW:CGCGCCCCCGGTTTCTAT, RV:CCTTCCCCATGGTGTCTGAGC).

### 2.5 Differentiation protocols

Mesoderm [7]: H1 cells were propagated for 72 hrs in 6 well plates. At that point, ActA-mTeSR or TGFβ-mTeSR was substituted with RPMI 1640 (Gibco; 21,875,034) containing 1% Glutamax (Gibco; 35,050,061), 1% MEM Non-Essential Amino Acids (Gibco; 11,140,050), 1% Penicillin-Streptomycin (Gibco; 15,070,063), 1% Insulin-Transferrin-Selenium (Gibco; 41,400,045), 0.1 mM β-mercaptoethanol (Gibco; 31,350,010),  50 ng/ml Activin-A (Dr Marko Hyvönen, University of Cambridge) and 50 ng/ml BMP4 (Gibco; PHC9534).

Endoderm [8]: H1 cells were propagated for 72 hrs in 6 well plates. At that point, ActA-mTeSR or TGFβ-mTeSR was substituted with RPMI 1640 (Gibco; 21,875,034) containing 1% Glutamax (Gibco; 35,050,061), 1% Penicillin-Streptomycin (Gibco; 15,070,063), 0.5% Fetal Bovine Serum (Gibco; 16,000,044) and 100 ng/ml Activin A (Dr Marko Hyvönen, University of Cambridge). At 24hr, medium was replenished and at 72hr FBS concentration was increased to 2%. Total RNA was isolated 5 days after induction.

Ectoderm [9]: H1 cells were seeded on non-adherent plates in DMEM/F-12 (Gibco; 12,634,010) containing 20% KnockOut Serum Replacement (Gibco; A3181502), 1% MEM Non-Essential Amino Acids (Gibco; 11,140,050), 1% l-Glutamine (Gibco; 25,030,149) and 0.1 mM β-mercaptoethanol (Gibco; 31,350,010). Suspension conditions enabled the folding of colonies and the subsequent formation of embryoid bodies. At 96hr, cell aggregates were transferred in DMEM/F-12 (Gibco; 12,634,010) containing 1% MEM Non-Essential Amino Acids (Gibco; 11,140,050) and 2 μg/ml Heparin (Gibco; RP-43,138). After 7 days, embryoid bodies were transferred to 35 mm μ-dishes (IB81151; Ibidi) that were pre-coated with laminin (Gibco; 23,017,015). On day 10 cells were fixed with 4% PFA.

### 2.6 Karyotype

Sub-confluent H1 cells cultured in ActA- or TGFβ-mTeSR were treated with 0,04ug/ml Karyomax colcemid (Gibco; 15,212) for 2 hrs at 37 °C. Next, cells were dissociated with trypsin, spun down and resuspended in a hypotonic KCL solution (0,56% w/v) for 30 min at 37 °C. Cells were spun down, resuspended in 2 ml KCL and fixed by applying an ice-cold solution of methanol/acetic acid (3:1), dropwise. Fixed cells were kept at −20 °C overnight and processed for Giemsa staining.

### 2.7 Teratoma formation assay

Sub-confluent H1 cells cultured in ActA- or TGFβ-mTeSR were manually dissected with an insulin needle and digested with 1 mg/ml dispase (Gibco; 17,105–041). The cell clumps were spun down for 3 min at 100 g and resuspended in 150μl matrigel. The mixture was injected through a 25 G 7/8 needle (BD Biosciences; 305,124) subcutaneously into the hind legs of immunodeficient NOD/SCID mice. Prior to injection, the matrigel, tips and syringes were stored on ice to avoid matrix polymerization. Mice were maintained under pathogen free conditions, at the animal house of the Biomedical Research Foundation (Academy of Athens, Greece). 10 weeks later, animals were sacrificed and the resulting teratomas were fixed, embedded in paraffin, dissected and stained with haematoxylin/eosin.

## Ethics Statement

All animal experimentation was performed in accordance with directive 2010/63/EU and the Amsterdam protocol on animal protection and welfare. Permission for the use of H1 embryonic stem cells was obtained from the Steering Committee for the UK Stem Cell Bank and Use of Human Stem Cells, MRC, UK.

## CRediT Author Statement

Conceptualization AP, VC, TF, CM; Methodology AP, VC; Validation AP, VC; Formal Analysis AP, VC; Investigation AP, VC, DS; Resources AP, VC, MH, CM; Data Curation AP, VC; Writing-Original Draft AP, VC; Writing-Review & Editing AP, VC, MH,TF, CM; Visualization AP, VC; Supervision TF, CM; Administration CM; Funding Acquisition CM.

## Declaration of Competing Interest

The authors declare that they have no known competing financial interests or personal relationships which have or could be perceived to have influenced the work reported in this article.
